# Surgical outcomes of hepatocellular carcinoma with extrahepatic bile duct tumor thrombus: a multicenter study

**DOI:** 10.3389/fonc.2023.1291479

**Published:** 2023-12-04

**Authors:** Li-Ming Huang, Zhen-Xin Zeng, Jun-Yi Wu, Yi-Nan Li, Jin-Xiu Wang, Yang-Kai Fu, Jia-Yi Wu, Shao-Ming Wei, Jia-Hui Lv, Wei-Zhao Chen, Rong-Fa Huang, Shu-Qun Cheng, Mao-Lin Yan

**Affiliations:** ^1^ Shengli Clinical Medical College, Fujian Medical University, Fuzhou, Fujian, China; ^2^ Department of Hepatobiliary Pancreatic Surgery, Fujian Provincial Hospital, Fuzhou, Fujian, China; ^3^ Department of General Surgery, Fujian Provincial Hospital South Branch, Fuzhou, Fujian, China; ^4^ Department of Hepatic Surgery VI, Eastern Hepatobiliary Surgery Hospital, Second Military Medical University, Shanghai, China

**Keywords:** hepatocellular carcinoma, hepatectomy, bile duct resection, R0 resection, obstructive jaundice, major vascular invasion, overall survival, recurrence-free survival

## Abstract

**Background:**

The long-term prognosis after surgery of patients with hepatocellular carcinoma (HCC) and extrahepatic bile duct tumor thrombus (Ex-BDTT) remains unknown. We aimed to identify the surgical outcomes of patients with HCC and Ex-BDTT.

**Methods:**

A total of 138 patients with Ex-BDTT who underwent hepatectomy with preservation of the extrahepatic bile duct from five large hospitals in China between January 2009 and December 2017 were included. The Cox proportional hazards model was used to analyze overall survival (OS) and recurrence-free survival (RFS).

**Results:**

With a median follow-up of 60 months (range, 1–127.8 months), the median OS and RFS of the patients were 28.6 and 8.9 months, respectively. The 1-, 3-, and 5-year OS rates of HCC patients with Ex-BDTT were 71.7%, 41.2%, and 33.5%, respectively, and the corresponding RFS rates were 43.5%, 21.7%, and 20.0%, respectively. Multivariate analysis identified that major hepatectomy, R0 resection, and major vascular invasion were independent prognostic factors for OS and RFS. In addition, preoperative serum total bilirubin ≥ 4.2 mg/dL was an independent prognostic factor for RFS.

**Conclusion:**

Major hepatectomy with preservation of the extrahepatic bile duct can provide favorable long-term survival for HCC patients with Ex-BDTT.

## 1 Introduction

Liver cancer is the fourth most common cause of cancer deaths worldwide, of which approximately 75%–85% are hepatocellular carcinomas (HCCs) (1). Tumor cells invade the portal vein and its branches and can form a portal vein tumor thrombus, which has been demonstrated as a poor prognostic factor (2, 3). Similarly, HCC can spread into the bile duct and can cause a bile duct tumor thrombus (BDTT). Based on the location of the BDTT in the biliary tree, it can be categorized as intrahepatic or extrahepatic BDTT (Ex-BDTT). The latter can cause extrahepatic bile duct obstruction and cause hemobilia, cholangitis, obstructive jaundice, and other complications (4–6).

Surgical excision has been proven to be the first choice for treating HCC with BDTT in some studies (7–11). However, due to the low incidence rate, only a few studies focusing on a small number of patients have shown concern about this issue. There is no consensus on the best surgical methods for treating HCC with BDTT, especially for Ex-BDTT. Some studies have shown that extrahepatic bile duct resection (BDR) can decrease the local residual and recurrence rates (7, 8, 10). In contrast, other studies have shown that most extrahepatic bile ducts should be preserved because Ex-BDTT seldom invades the extrahepatic bile duct wall (9, 12–14). Therefore, the long-term prognosis after surgery of patients who underwent Ex-BDTT remains unknown. In this study, all cases collected postoperative pathological data, and all patients were diagnosed with HCC with BDTT by postoperative pathology.

The purpose of this study was to determine the surgical results of hepatectomy with preservation of extrahepatic bile ducts in patients with HCC and Ex-BDTT.

## 2 Materials and methods

### 2.1 Patients

We collected the medical information of 138 patients with Ex-BDTT, who underwent hepatectomy with preservation of the extrahepatic bile duct, in five large hospitals in China between January 2009 and December 2017.

During this period, the total number of HCC patients in these institutions was 8815, and the number of HCC with BDTT patients was 442. These institutions included Fujian Provincial Hospital (Fuzhou, China), First Affiliated Hospital of Fujian Medical University (Fuzhou, China), Eastern Hepatobiliary Surgery Hospital of Second Military Medical University (Shanghai, China), West China Hospital of Sichuan University (Chengdu, China), and Zhongshan Hospital of Xiamen University (Xiamen, China). The original data were collected and then merged for analysis. The Institutional Review Committee of each cooperative hospital reviewed and approved our study. The patients themselves or their authorized persons signed a written informed consent to use their data for research purposes. This retrospective study did not contradict the principles of the Declaration of Helsinki.

The collected data included demographic, perioperative information, histopathological information, recurrence, and survival data. The diagnosis of Ex-BDTT was defined as tumor thrombus in the extrahepatic bile duct, which is detected by preoperative clinical imaging examination (such as computed tomography [CT], magnetic resonance cholangiopancreatography [MRCP] or magnetic resonance imaging [MRI]) and intraoperative findings, and diagnosed by postoperative pathological reports. The tumor thrombus invading the first-order branch or main trunk of the portal and/or hepatic vein can be detected by CT or MRI preoperatively. This is called a major vascular invasion (MVI). The greatest dimension of the largest lesion is regarded as the tumor size regardless of whether there are multiple tumors. The staging system was based on the American Joint Committee on Cancer (AJCC) 8th edition (15). The definition of post-hepatectomy liver failure (PHLF) and the classification of postoperative complications were carried out according to the recommendations of the International Study Group of Liver Surgery and the Clavien-Dindo classification (16, 17).

The inclusion criteria were (1) surgical resection and (2) HCC with Ex-BDTT confirmed by histopathological examination. The exclusion criteria were (1) Hepatectomy with BDR (n = 18), (2) recurrent tumor (n = 3), (3) preoperative anticancer treatment (n = 2), (4) history of other cancers (n = 2), (5) Child-Pugh grade C (n = 2), (6) poorly tolerated hepatectomy (n = 1) and (7) incomplete data (n = 5). The flow chart for screening all cases of HCC with Ex-BDTT is shown in **Supplementary Figure S1**.

### 2.2 Preoperative management

There is no unified standard for biliary drainage in this study. The level of serum total bilirubin (TBil) in all patients with biliary drainage is greater than 11.7 mg/L. A percutaneous transhepatic cholangial drainage tube is usually placed in the remnant intrahepatic bile duct of the liver. Before surgery, CT or MRI was used to evaluate the patient’s future liver reserve (FLR). Major hepatectomy was indicated only when the residual liver volume of patients with liver cirrhosis reduces to 40% of the liver parenchymal volume. Preoperative liver function evaluation was comprehensively evaluated by each center.

### 2.3 Surgical methods

In this study, major hepatectomy was defined as liver resection greater than or equal to hemihepatectomy, including central hepatectomy (which greater than or equal to 3 liver segments). Negative bile duct and specimen margins on histology, no evidence of tumor recurrence on CT or MRI investigations, and normal serum alpha-fetoprotein (AFP) levels defined R0 resection at 90 days postoperatively (8).

Tumor size, the number of tumors, tumor position, and hepatic functional reserve were used to evaluate the extent of hepatectomy. The Pringle maneuver was performed, if necessary. Extrahepatic bile duct was preserved unless the Ex-BDTT could not be removed through thrombectomy or had invaded the extrahepatic bile duct wall. This was a multicenter retrospective study, and the surgical methods of each center were different. However, most of the tumor thrombectomy techniques in each center were similar to the “peeling-off technique” of Yamamoto et al. (12). We incised the bile duct, peeled off the BDTT, confirmed that the margin of the bile duct was negative by intraoperative frozen section analysis, and sutured the bile duct incision with 5-0 or 6-0 absorbable monofilament thread. In the Fujian Provincial Hospital, the tumor thrombectomy technique, called “q-shaped thrombectomy”, which we have previously reported, was also used in some patients (18). Moreover, choledochoscopy was performed routinely during the operation to confirm whether the tumor thrombus was removed neatly.

### 2.4 Postoperative follow-up and management of recurrence

The first follow-up was conducted in the first month after the operation, every three months in the first year, and every six months thereafter until death. Laboratory examinations (including AFP, liver function, etc.) and imaging examinations (including CT or MRI enhancement, etc.) were performed at each re-examination. Recurrence was treated using a multidisciplinary approach that included surgical resection, systemic therapy, radiofrequency ablation, or transarterial chemoembolization, according to the recurrence site and liver functional reserves. The time between the date of the first curative hepatectomy and the date of death or last follow-up is called overall survival (OS). The time between the date of the first curative hepatectomy and the date of the first relapse or last follow-up is called recurrence-free survival (RFS). The last follow-up ended on June 1, 2021.

### 2.5 Statistical analysis

Continuous data are expressed as medians with ranges. Categorical variables are expressed as counts with percentages (%). The Kaplan–Meier method was used to calculate the rates of OS and RFS, and the log-rank test was used for comparison of survival between groups. The Kaplan–Meier curves were produced by R software (R Foundation for Statistical Computing, Vienna, Austria). The proportional hazards assumption was assessed by performing Schoenfeld’s tests and plots and was met for all analyses. The Cox proportional hazards regression model was performed for univariate and multivariate analyses. A two-tailed p-value of < 0.05 was considered statistically significant. IBM SPSS (version 24.0; IBM Corp, Armonk, NY, USA) was used for statistical analysis.

## 3 Results

### 3.1 Patient characteristics

One hundred and thirty-eight HCC patients with Ex-BDTT were included in the present study. The clinicopathological features of patients having HCC with Ex-BDTT are presented in **Table 1**. At the first visit, 94 patients (68.1%) had obstructive jaundice, and 27 (19.6%) received preoperative biliary drainage. Among them, twenty-two patients (15.9%) underwent percutaneous transhepatic cholangial drainage, and five patients (3.6%) underwent endoscopic retrograde cholangiopancreatography. The level of TBil in all patients with biliary drainage is greater than 11.7 mg/L. In the biliary drainage group, the median serum TBil decreased from 17.5 mg/dl to 7.4 mg/dl. Twenty-six patients (18.8%) presented with MVI preoperatively. Sixty-five patients (47.1%) underwent major hepatectomy and 103 patients (74.6%) underwent R0 resection. The R0 resection rates were 53.4% and 46.6% in those who underwent major hepatectomy and those who did not, respectively (p = 0.011). Liver cirrhosis was confirmed in 74 patients (53.6%). According to the Clavien-Dindo classification of postoperative complications, 12 patients (8.7%) were classified as having grade IIIb-V. Sixty-one (44.2%) patients underwent transarterial chemoembolization, which is the most common postoperative adjuvant treatment.

**Table 1:** Clinicopathological features and operative procedures.

Variables	Values (n = 138)
Age (years)	52 (18–74)
Male	122 (88.40%)
ALB (g/L)	38.8 (21.2–47.3)
INR	1.05 (0.89–1.26)
AFP ≥ 400 (ng/mL)	69 (50.0%)
HBsAg (positive)	108 (78.3%)
Obstructive jaundice	94 (68.1%)
TBil on admission (mg/dL)	4.6 (0.5–25.7)
Pre-operative TBil (mg/dL)	4.2 (0.5–11.7)
Biliary drainage	
PTCD	22 (15.9%)
ERCP	5 (3.6%)
None	111 (80.4%)
TBil in biliary drainage group (mg/dL)	
Pre- drainage TBil	17.5 (11.7–24.7)
Post-drainage TBil	7.4 (2.2–14.7)
Child–Pugh class B	81 (58.7%)
Major hepatectomy	65 (47.1%)
R0 resection	103 (74.6%)
Tumor size ≥ 5cm	80 (58.0%)
Multiple tumors	40 (29.0%)
Poor differentiation	36 (26.1%)
Liver cirrhosis	74 (53.6%)
Microvascular invasion	60 (43.5%)
Major vascular invasion	26 (18.8%)
8^th^ AJCC Stage	
IA	8 (5.8%)
IB	62 (44.9%)
II	21 (15.2%)
IIIA	21 (15.2%)
IIIB	26 (18.8%)
Surgical duration (min)	195 (120–420)
Blood loss (ml)	400 (50–2000)
Postoperative hospital stay (days)	12 (4–58)
90-day mortality	5 (3.6%)
PHLF grade	
A	2 (1.5%)
B	2 (1.5%)
C	2 (1.5%)
Clavien-Dindo classification (IIIb-V)	12 (8.7%)
Post-operative adjuvant treatment	
TACE	61(44.2%)
Chemotherapy/Radiotherapy	7 (2.6%)
Multimodality therapy	10 (7.2%)
Symptomatic support treatment	60(43.5%)

HCC, hepatocellular carcinoma; Ex-BDTT, extrahepatic bile duct tumor thrombus; ALB, serum albumin; INR, international normalized ratio; AFP, serum alpha-fetoprotein; HBsAg, hepatitis B surface antigen; TBil, total bilirubin; PTCD, percutaneous transhepatic cholangial drainage; ERCP, endoscopic retrograde cholangiopancreatography; BDR, bile duct resection; AJCC, American Joint Committee on Cancer; PHLF, post-hepatectomy liver failure; TACE, transcatheter arterial chemoembolization.

### 3.2 Overall survival and recurrence-free survival

**Figure 1:**
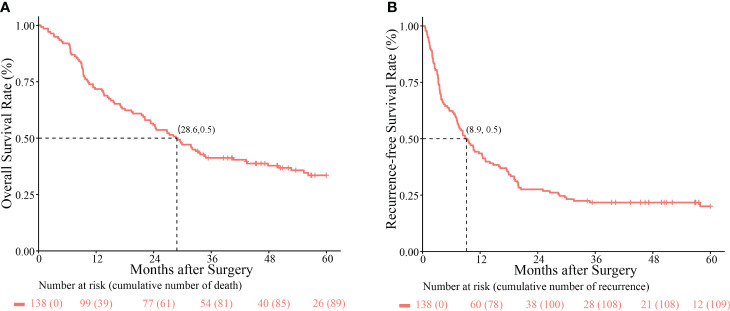
Kaplan–Meier survival curves comparing overall survival **(A)** and recurrence-free survival rates **(B)** among hepatocellular carcinoma patients with extrahepatic bile duct tumor thrombus who underwent hepatectomy.

The median follow-up of 60 months (range, 1–127.8 months) and the median OS and RFS of the patients with Ex-BDTT were 28.6 and 8.9 months, respectively. The 1-, 3-, and 5-year OS rates of patients with HCC having Ex-BDTT were 71.7%, 41.2%, and 33.5%, respectively (**Figure 1A**), and the RFS rates were 43.5%, 21.7%, and 20.0%, respectively (**Figure 1B**).

**Figure 2:**
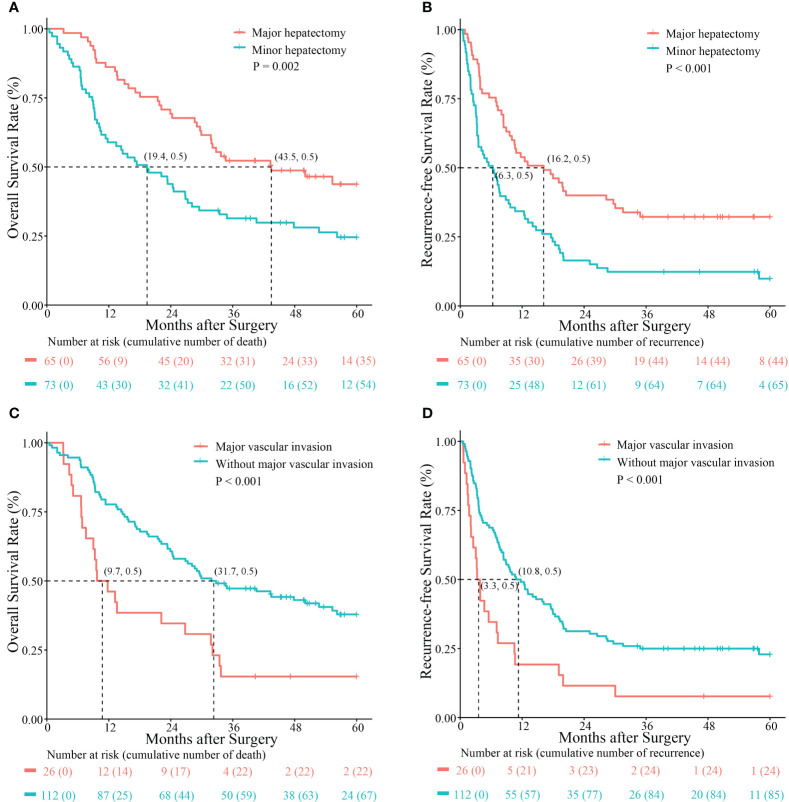
Kaplan–Meier survival curves comparing overall survival and recurrence-free survival rates between hepatocellular carcinoma patients with extrahepatic bile duct tumor thrombus who underwent major hepatectomy and those without major hepatectomy **(A, B)** and those with and without major vascular invasion **(C, D)**.

As shown in **Figure 2**, better OS and RFS rates were observed following major hepatectomy. The 1-, 3-, and 5-year OS rates in patients with Ex-BDTT who underwent major hepatectomy and those who did not were 86.2%, 52.3%, and 43.8%, versus 58.9%, 31.4%, and 24.6%, respectively (p = 0.002) (**Figure 2A**). The 1-, 3-, and 5-year RFS rates for those who underwent major hepatectomy and those who did not were 58.9%, 32.2%, and 32.2% versus 34.2%, 12.3%, and 9.9%, respectively (p < 0.001) (**Figure 2B**). Moreover, the 1 -, 3 - and 5-year OS rates of patients with MVI (46.2%, 15.4%, and 15.4%, respectively) were significantly lower than those without MVI (77.7%, 47.3%, and 37.9%, respectively) (p = 0.001) (**Figure 2C**). Similarly, the 1-, 3-, and 5-year RFS rates of patients with MVI (19.2%, 7.7%, and 7.7%, respectively) were also significantly lower than those without MVI (49.1%, 25.0%, and 22.9%, respectively) (p < 0.001) (**Figure 2D**).

### 3.3 Risk factors for overall survival and recurrence-free survival

As seen in **Table 2**, the results of the Cox multivariate analysis indicated that major hepatectomy, R0 resection, and MVI were independent prognostic factors for OS (hazard ratio [HR] 0.452, 95% confidence interval [CI] 0.295–0.695, p < 0.001; HR 0.292, 95% CI 0.185–0.460, p < 0.001; and HR 2.010, 95% CI 1.195–3.379, p = 0.008, respectively) and RFS (HR 0.518, 95% CI 0.344–0.779, p = 0.002; HR 0.176, 95% CI 0.108–0.287, p < 0.001; and HR 1.781, 95% CI 1.073–2.954, p = 0.026, respectively). Preoperative serum TBil ≥ 4.2 mg/dL was an independent prognostic factor for RFS (HR 1.803, 95% CI 1.204–2.700; p = 0.004).

**Table 2:** Prognostic factors for overall survival and recurrence-free survival.

Variables	Overall survival				Recurrence-free survival			
	Univariate analysis		Multivariate analysis		Univariate analysis		Multivariate analysis	
	HR (95% CI)	p-value	HR (95% CI)	p-value	HR (95% CI)	p-value	HR (95% CI)	p-value
Age ≥ 65 years	0.718 (0.360–1.431)	0.346			0.678 (0.372–1.236)	0.204		
Male	0.912 (0.472–1.761)	0.784			1.418 (0.739–2.722)	0.294		
Preoperative TBil ≥ 4.2 mg/dL	1.328 (0.874–2.017)	0.184			1.531 (1.050–2.233)	0.027	1.803 (1.204–2.700)	0.004
ALB ≥ 35 g/L	0.521 (0.310–0.876)	0.014	0.894 (0.500–1.599)	0.706	0.725 (0.436–1.204)	0.214		
INR ≥ 1.12	1.228 (0.636–2.373)	0.540			0.971 (0.520–1.811)	0.925		
AFP ≥ 400 ng/mL	1.180 (0.778–1.789)	0.436			1.208 (0.829–1.760)	0.325		
Child–Pugh class B	1.215 (0.794–1.859)	0.370			1.514 (1.031–2.223)	0.034	0.616 (0.333–1.137)	0.121
HBsAg (positive)	1.584 (0.921–2.724)	0.096			1.374 (0.861–2.194)	0.183		
Tumor size ≥ 5 cm	1.080 (0.733–1.589)	0.973			1.220 (0.833–1.787)	0.308		
Multiple tumors	0.903 (0.598–1.366)	0.631			1.035 (0.680–1.576)	0.873		
Major hepatectomy	0.514 (0.336–0.788)	0.002	0.452 (0.295–0.695)	<0.001	0.493 (0.336–0.723)	<0.001	0.518 (0.344–0.779)	0.002
R0 resection	0.251 (0.161–0.392)	<0.001	0.292 (0.185–0.460)	<0.001	0.170(0.110–0.262)	<0.001	0.176 (0.108–0.287)	<0.001
Poor differentiation	1.000 (0.626–1.597)	1.000			1.120 (0.752–1.730)	0.611		
Liver cirrhosis	1.135 (0.747–1.724)	0.554			1.045 (0.719–1.520)	0.817		
Microvascular invasion	1.540 (1.015–2.337)	0.042	1.177 (0.728–1.904)	0.506	1.315 (0.901–1.920)	0.156		
Major vascular invasion	2.295 (1.411–3.732)	0.001	2.010 (1.195–3.379)	0.008	2.114 (1.339–3.336)	0.001	1.781 (1.073–2.954)	0.026

HCC, hepatocellular carcinoma; Ex-BDTT, extrahepatic bile duct tumor thrombus; HR, hazard ratio; CI, confidence interval; TBil, total bilirubin; ALB, serum albumin; INR, international normalized ratio; AFP, serum alpha-fetoprotein; HBsAg, hepatitis B surface antigen.

### 3.4 Perioperative outcomes and recurrence sites after hepatectomy

As shown in **Table 3**, There was no significant difference between the two groups in terms of surgical duration, blood loss, postoperative hospital stay, 90-day mortality, PHLF, and postoperative complications. Meanwhile, the surgical duration of major hepatectomy is longer than that of minor hepatectomy, and the difference is statistically significant (p < 0.001). However, there was no significant difference in the other observation indicators. Recurrence sites after hepatectomy are described in **Table 4**. Intrahepatic recurrence is the most common of all recurrent sites (58.2%; 64/110).

**Table 3:** Perioperative outcomes of obstructive jaundice group and without obstructive jaundice group, major hepatectomy group and minor hepatectomy group.

Variable	Without obstructive jaundice (n = 44)	Obstructive jaundice (n = 94)	p-value	Minor hepatectomy (n = 73)	Major hepatectomy (n = 65)	p-value
Surgical duration (min)	199 (130–373)	195 (120–420)	0.357	188 (130-275)	210 (120-420)	< 0.001
Blood loss (ml)	300 (100–600)	400 (50–2000)	0.258	300 (50-1800)	400 (100-2000)	0.303
Postoperative hospital stay (days)	12 (4–27)	12 (6–58)	0.082	12 (4-58)	12 (5-28)	0.351
PHLF			0.664			0.490
Yes	1	5		4	2	
No	43	89		69	63	
90-day mortality			1.000			0.060
Yes	1	4		5	0	
No	43	90		68	65	
Clavien-Dindo classification			1.000			0.693
I-IIIa	40	86		66	60	
IIIb-V	4	8		7	5	

HCC, hepatocellular carcinoma; Ex-BDTT, extrahepatic bile duct tumor thrombus; PHLF, post-hepatectomy liver failure.

**Table 4:** Recurrence pattern.

Location of Recurrence	Values (n = 110)
Intrahepatic	64 (58.2%)
Extrahepatic	10 (9.1%)
Intra - and extrahepatic	4 (3.6%)
Intrahepatic and intrahepatic bile duct	21 (19.1%)
Local BDTT^#^	11 (10%)

HCC, hepatocellular carcinoma; Ex-BDTT, extrahepatic bile duct tumor thrombus. ^#^It refers to the tumor recurrence site was on the original BDTT site.

## 4 Discussion

HCC with BDTT can be classified into different types based on the extent of tumor thrombus invasion into the bile duct. Cheng et al. proposed a classification: Type I: intrahepatic BTT; and Type II: extrahepatic BTT involving a common bile duct or common hepatic duct (19). This study referred to the classification proposed by Cheng et al., where the EX-BDTT is equivalent to Type II. HCC presenting with obstructive jaundice due to BDTT, especially Ex-BDTT, has been classified as “icteric-type hepatoma” (20). Hyperbilirubinemia increases postoperative complications, such as PHLF and intraoperative bleeding, and leads to a poor prognosis (4–6, 21). This is probably ascribed to the insufficient liver reserve caused by obstructive jaundice. Thus, HCC patients with jaundice caused by advanced tumor infiltration, advanced liver cirrhosis, or progressive end-stage liver failure have no chance of achieving remission or cure. However, patients with HCC having Ex-BDTT often have reversible hyperbilirubinemia and hypoalbuminemia, which is caused by biliary obstruction. Previous research reported that biliary drainage procedures, including percutaneous transhepatic cholangial drainage and endoscopic retrograde cholangiopancreatography, are beneficial to the prognosis of patients with HCC having obstructive jaundice (9, 10). Preoperative biliary drainage can improve coagulation function, liver function, and reduce postoperative complications (10, 22–26). However, there is a debate about whether the liver function of patients with jaundice is reduced to a normal level before surgery. Some scholars have advocated reducing serum TBil to < 2 mg/dL before surgery (9–11). In our data, the serum TBil in the biliary drainage group did not decrease to normal levels. The median serum TBil in the biliary drainage group decreased from 17.5 mg/dL to 7.4 mg/dL. However, the 5-year OS rate in our research was almost the same as in the previous study in which TBil was reduced to 2 mg/L (9–11). In addition, it did not increase complications, such as PHLF and intraoperative bleeding. We also found that preoperative serum TBil ≥ 4.2 mg/dL was an independent prognostic factor for RFS, and reducing serum TBil to < 4.2 mg/dL before surgery was also feasible. However, the optimal TBil level before surgery requires further investigation.

Several studies have emphasized the importance of major hepatectomy for HCC with BDTT (10, 13, 27). Major hepatectomy can reduce the tumor residual in the extrahepatic bile ducts and the liver parenchyma. In addition, it could achieve anatomic resection to increase the R0 resection rate (10, 28, 29). Although some studies reported that major hepatectomy was associated with relatively high postoperative mortality, others indicated that major hepatectomy could reduce the risk of PHLF in HCC patients with adequate FLR (30–32). In the present study, major hepatectomy was an independent favorable prognostic factor for both OS and RFS. In addition, major hepatectomy did not increase postoperative complications, such as PHLF compared with the minor hepatectomy group, because all patients in the major hepatectomy group had sufficient FLR. Therefore, we believe that major hepatectomy is safe and feasible for HCC patients with BDTT when the liver function and FLR are carefully evaluated before surgery. However, it still needs further research.

The preservation of the extrahepatic bile duct for Ex-BDTT cases is still inconclusive. These controversies are possibly owing to the low incidence of BDTT and the lack of randomized controlled trials. Several studies have supported the use of hepatectomy with BDR, which can avoid residual tumors in the extrahepatic bile ducts and reduce postoperative recurrences (7, 8, 10, 27, 33). However, we adopted major hepatectomy with preservation of the extrahepatic bile duct in the present study. First, BDTT rarely invades the extrahepatic bile duct walls and can be easily peeled off (12–14, 34, 35). Second, bile duct recurrence lesions can be successfully treated by regional treatment, and BDR is a risk factor for liver abscesses after regional therapy due to retrograde infection through cholangiojejunostomy (12, 36–40). In the current research, the 5-year OS and RFS rates of Ex-BDTT patients who underwent major hepatectomy were similar to those of patients who underwent BDR in other studies (45.1% and 33.0%) (33). Therefore, preservation of extrahepatic bile ducts combined with major hepatectomy can be recommended in patients with Ex-BDTT, unless the BDTT cannot be removed by tumor thrombectomy or it invades the extrahepatic bile duct wall.

Although the number of Ex-BDTT cases in our study was the largest worldwide, there were some limitations. This was a retrospective study and lacked a control group (patients with HCC but without BDTT). In the future, well-designed multicenter randomized controlled trials are needed for further investigation. Moreover, the present study was carried out in China, which has a high incidence rate of hepatitis B virus-related HCC. Thus, these results may not be applicable to patients with HCC infected with other hepatitis viruses.

In conclusion, for patients with HCC having Ex-BDTT, major hepatectomy with preservation of the extrahepatic bile duct can provide an opportunity for favorable long-term survival.

## Data availability statement

The raw data supporting the conclusions of this article will be made available by the authors, without undue reservation.

## Ethics statement

The studies involving humans were approved by The Institutional Review Board of Fujian Provincial Hospital. The studies were conducted in accordance with the local legislation and institutional requirements. The participants provided their written informed consent to participate in this study.

## Author contributions

LH: Conceptualization, Data curation, Investigation, Validation, Writing – original draft. ZZ: Conceptualization, Data curation, Investigation, Writing – original draft, Visualization. JuW: Data curation, Investigation, Writing – original draft. YL: Data curation, Investigation, Writing – original draft. JW: Data curation, Investigation, Visualization, Writing – review & editing. YF: Data curation, Investigation, Visualization, Writing – review & editing. JiW: Investigation, Writing – review & editing, Project administration. SW: Investigation, Project administration, Writing – review & editing. JL: Investigation, Project administration, Writing – review & editing. WC: Investigation, Project administration, Writing – review & editing. RH: Investigation, Project administration, Writing – review & editing. SC: Writing – review & editing, Conceptualization, Resources, Supervision. MY: Conceptualization, Resources, Supervision, Writing – review & editing, Funding acquisition.

## Supplementary material

Supplementary Figure 1 Flowchart for screening all HCC with Ex-BDTT cases. HCC, hepatocellular carcinoma; Ex-BDTT, extrahepatic bile duct tumor thrombus; BDR, bile duct resection.
