# Supplementary figures and images for: Surgical outcomes of hepatocellular carcinoma with extrahepatic bile duct tumor thrombus: a multicenter study
Source: Front Oncol. 2023 Dec 4;13:1291479. doi: 10.3389/fonc.2023.1291479 (PMC10726108; doi:10.3389/fonc.2023.1291479)

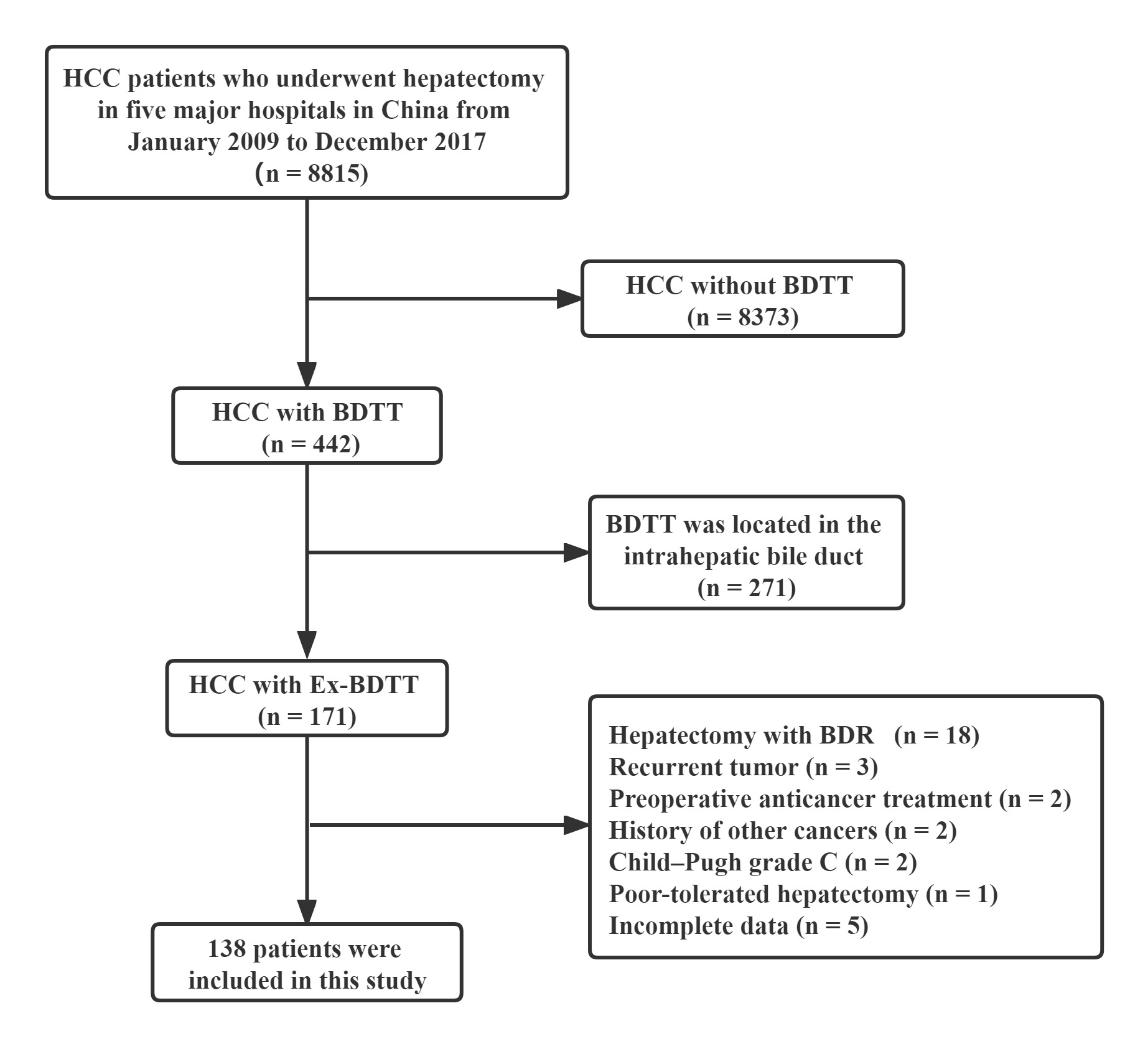

Supplement: Supplementary Figure 1 — Flowchart for screening all HCC with Ex-BDTT cases. HCC, hepatocellular carcinoma; Ex-BDTT, extrahepatic bile duct tumor thrombus; BDR, bile duct resection. [file Image_1.jpeg]
